# OA03.01. Acupuncture for chronic pain: an individual patient data meta-analysis of randomized trials

**DOI:** 10.1186/1472-6882-12-S1-O9

**Published:** 2012-06-12

**Authors:** A Vickers, A Cronin, A Maschino, G Lewith, H MacPherson, N Victor, N Foster, K Sherman, C Witt, K Linde

**Affiliations:** 1 Memorial Sloan-Kettering Cancer Center, New York City, USA; 2 University of Southampton, Southhampton, United Kingdom; 3 University of York, York, United Kingdom; 4 University of Heidelberg, Heidelberg, Germany; 5 University of Keele, Keele, United Kingdom; 6 Group Health Cooperative, Seattle, USA; 7 Charite University, Berlin, Germany; 8 Technical University of Munich, Munich, Germany

## Purpose

Although acupuncture is widely used for chronic pain, there remains uncertainty about the clinical impact. The aim of this study is to determine the effect size of acupuncture vs. sham acupuncture and the effect size of acupuncture vs. non acupuncture controls for four conditions, back and neck pain, osteoarthritis, and chronic headache. More precision can be obtained in a meta-analysis when using individual patient data, and it also provides an opportunity to explore treatment effect modifiers.

## Methods

Data sources included MEDLINE, the Cochrane Collaboration and the citation lists of systematic reviews. Studies were included if randomized with unambiguously adequate allocation concealment. Individual patient data was obtained for 29 of 31 eligible trials, with a total of 17,922 patients, and analyzed in a single database.

## Results

Patients receiving acupuncture had less pain than those receiving sham acupuncture for back and neck pain, osteoarthritis, and chronic headache (all p<0.001). After exclusion of an outlying set of trials showing very large differences between acupuncture and sham, the effect size for acupuncture was similar across the pain conditions, [SMD -0.23 (95% CI -0.33, -0.13)], [SMD -0.16 (-0.25, -0.07)] , and [SMD -0.15 (-0.24, -0.07)] (all p<0.001) respectively. Larger effect sizes were found when comparing acupuncture to non acupuncture controls (p<0.001). Acupuncture had a more beneficial effect in patients with worse baseline pain scores and worse baseline mental status (p<0.005).

## Conclusion

Individual patient data meta-analysis is a powerful method of evaluating effect sizes and exploring treatment effect modifiers. Our results from high quality trials show that acupuncture is effective for the treatment of chronic pain, with significant differences between true and sham acupuncture indicating that acupuncture is more than a placebo. However, these differences are relatively modest, suggesting that factors in addition to the specific effects of needling are important contributors to the therapeutic effects of acupuncture.

